# Hepatic p53 is regulated by transcription factor FOXO1 and acutely controls glycogen homeostasis

**DOI:** 10.1016/j.jbc.2022.102287

**Published:** 2022-07-20

**Authors:** Moritz Oster, Markus Galhuber, Jelena Krstic, Julia S. Steinhoff, Georgia Lenihan-Geels, Sascha Wulff, Marie F. Kiefer, Konstantin M. Petricek, Sylvia J. Wowro, Roberto E. Flores, Na Yang, Chen Li, Yueming Meng, Isabel Reinisch, Manuela Sommerfeld, Stefan Weger, Hansjörg Habisch, Tobias Madl, Tim J. Schulz, Andreas Prokesch, Michael Schupp

**Affiliations:** 1 Charité-Universitätsmedizin Berlin, Freie Universität Berlin, Humboldt-Universität zu Berlin, Institute of Pharmacology, Cardiovascular Metabolic Renal (CMR)-Research Center, Berlin, Germany; 2 Division of Cell Biology, Histology and Embryology, Gottfried Schatz Research Center for Cell Signaling, Metabolism & Aging, Medical University of Graz, Graz, Austria; 3 Department of Adipocyte Development and Nutrition, German Institute of Human Nutrition, Nuthetal, Germany; 4 Charité-Universitätsmedizin Berlin, Freie Universität Berlin, Humboldt-Universität zu Berlin, Institute of Virology, Campus Benjamin Franklin, Berlin, Germany; 5 Institute of Molecular Biology and Biochemistry, Medical University Graz, Graz, Austria; 6 German Center for Diabetes Research (DZD), München-Neuherberg, Germany; 7 Institute of Nutritional Science, University of Potsdam, Nuthetal, Germany

**Keywords:** p53, Foxo1, liver, glucose metabolism, glycogen, triglycerides, fasting, AAV, adeno-associated virus, ChIP, chromatin immunoprecipitation, FOXO1, forkhead box O1 protein, GYS, glycogen synthase, HFD, high-fat diet, *Igfbp1*, *insulin-like growth factor-binding protein 1*, NC, normal chow, *Pck1*, *phosphoenolpyruvate carboxykinase 1*, *Sesn2*, *sestrin 2*

## Abstract

The tumor suppressor p53 is involved in the adaptation of hepatic metabolism to nutrient availability. Acute deletion of p53 in the mouse liver affects hepatic glucose and triglyceride metabolism. However, long-term adaptations upon the loss of hepatic p53 and its transcriptional regulators are unknown. Here we show that short-term, but not chronic, liver-specific deletion of p53 in mice reduces liver glycogen levels, and we implicate the transcription factor forkhead box O1 protein (FOXO1) in the regulation of p53 and its target genes. We demonstrate that acute p53 deletion prevents glycogen accumulation upon refeeding, whereas a chronic loss of p53 associates with a compensational activation of the glycogen synthesis pathway. Moreover, we identify fasting-activated FOXO1 as a repressor of p53 transcription in hepatocytes. We show that this repression is relieved by inactivation of FOXO1 by insulin, which likely mediates the upregulation of p53 expression upon refeeding. Strikingly, we find that high-fat diet–induced insulin resistance with persistent FOXO1 activation not only blunted the regulation of p53 but also the induction of p53 target genes like p21 during fasting, indicating overlapping effects of both FOXO1 and p53 on target gene expression in a context-dependent manner. Thus, we conclude that p53 acutely controls glycogen storage in the liver and is linked to insulin signaling *via* FOXO1, which has important implications for our understanding of the hepatic adaptation to nutrient availability.

The transcription factor p53 is one of the most thoroughly investigated tumor suppressors and mutated in more than 50% of all human cancers (1, 2). As a unifying stress response of virtually all cell types, p53 is activated by a variety of stimuli such as genotoxic damage. The degree of p53 induction in a given cell type thereby depends on stress intensity and duration, leading to an adaptive response that involves cell-cycle arrest, senescence, and apoptosis, which are commonly referred to as classical p53 functions (3).

Initially, these responses were thought to occur primarily by inducing cell cycle inhibitors like *cyclin-dependent kinase inhibitor 1a* (*Cdkn1a*=*p21*). Further studies showed that p53’s tumor suppressor function is also dependent on its control of cellular metabolism (3). Accordingly, dysregulated energy homeostasis, such as oxygen or nutrient deprivation, potently induces p53 (4). These findings suggest that the tumor-suppressing and metabolic functions of p53 are interlinked and partially overlapping. Identifying novel regulators and functions of p53 in the context of glucose and fatty acid metabolism is therefore essential to understand the complex interactions between metabolic and oncologic diseases (5, 6).

We previously reported that fasting mice for 24 h induces a concerted upregulation of p53 target genes, including *p21*, in liver, muscle, and white adipose tissue (7). Consistently, and in line with many cancer cell types, exposing primary mouse and human hepatocytes to a low-glucose and serum-free starvation media robustly increased p53 protein expression (8). In *ad libitum*-fed adult mice, acute deletion of hepatic *Trp53* (*p53*) for only 96 h lowered glycogen and increased triglyceride levels in liver. Upon fasting, mice with liver-specific deletion of p53 became hypoglycemic (8). Thus, p53 is involved in the acute hepatic adaptation to nutrient withdrawal (9).

Whether liver-specific deletion of p53 induces long-term effects on glucose and fatty acid metabolism has not been determined. The transcriptional regulators that fine-tune hepatic *p53* expression are also unknown (8). We addressed these questions by characterizing a novel mouse model and found that acute but not chronic deletion of p53 in liver induces glycogen depletion and triglyceride accumulation. Acute deletion of p53 strongly impaired refeeding-induced glycogen accumulation in liver but elicited compensational activation of the glycogen synthesis pathway in the long term. Moreover, we found that p53 expression is transcriptionally induced by refeeding and insulin. This involved the fasting-activated transcription factor forkhead box O1 protein (FOXO1), which represses p53 in hepatocytes. High-fat diet (HFD) feeding blunted the transcriptional regulation of *p53* and of its target genes upon fasting, suggesting that FOXO1 interferes with hepatic p53 signaling in a complex manner. These findings have important implications for our understanding of the hepatic adaptations to nutrient availability.

## Results

### Hepatic p53 is expressed primarily in the nucleus and deleted efficiently by liver-specific Cre expression

**Figure 1:**
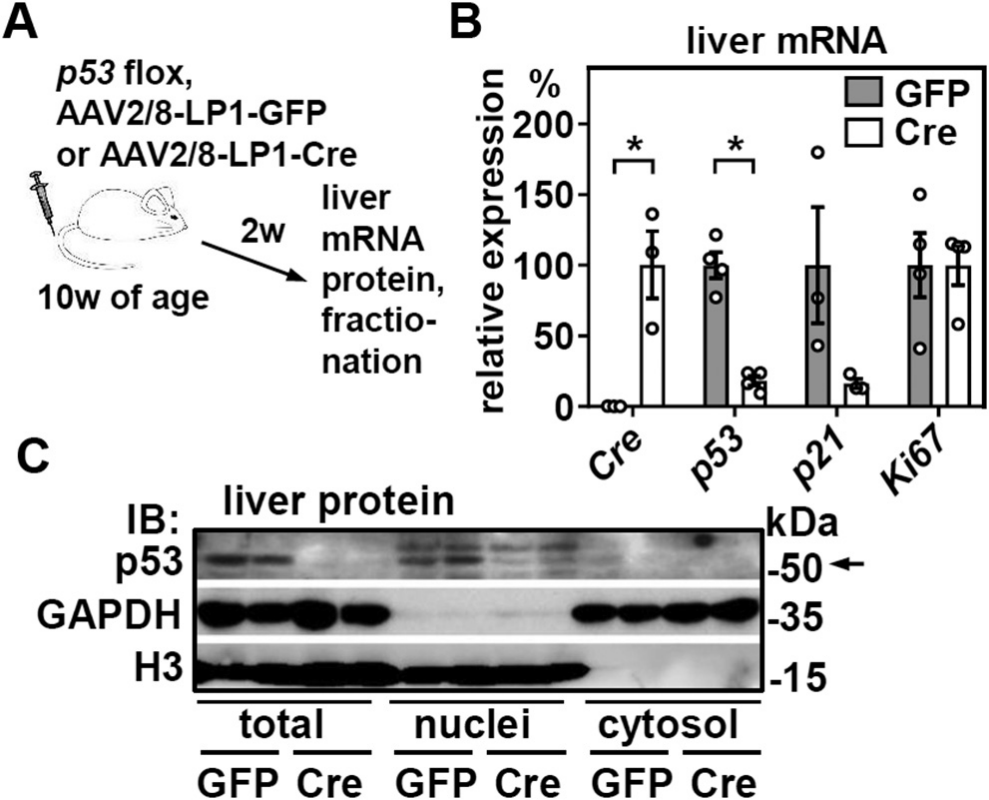
**Liver p53 is expressed primarily in the nucleus of hepatocytes and deleted efficiently by hepatocyte-specific AAV2/8-Cre expression in adult mice.***A*, male mice with floxed *p53* alleles were tail vein-injected with adeno-associated viruses (AAVs) serotype 2/8 expressing GFP or Cre under the control of the liver-specific LP1 promoter. *B*, 2 weeks later, mRNA expression of indicated genes was analyzed by qPCR. *C*, liver tissue was processed into nuclear and cytosolic fractions and p53 protein analyzed by immunoblotting. GAPDH and histone 3 (H3) served as loading and fractionation controls. In (*B*), data are represented as individual data points and mean ± SEM and ∗*p* < 0.05 *versus* mice with liver-specific GFP expression.

We tail vein–injected adult male mice homozygous for the floxed *p53* allele (10) with equal titers of adeno-associated viruses (AAVs), serotype 2/8, that express either GFP or Cre recombinase under the control of a synthetic and liver-specific LP1 promoter (11) (Fig. 1*A*). Tissue specificity of LP1-promoter driven AAV2/8 was validated by us previously and showed exclusive expression in liver but not in other organs that are readily infected by AAV2/8 in mice (12), including the brain (13). AAV’s deliver stable expression of the episomal transgene in liver for months and lack immunogenicity in mice (14), rendering them a superior tool to previously used adenoviruses (8). We found that hepatic Cre expression induced a robust deletion of p53 2 weeks after the injection (Fig. 1, *B* and *C*). This was mirrored by reduced expression of its target, *p21*, without inducing hepatocyte proliferation when assessed by *Ki67* as marker gene. Since Ki67 staining of liver sections was increased in our previous study after only 96 h of p53 deletion, the longer duration of p53 loss in this setting may induce compensatory regulations that normalize cell cycle progression and hepatocyte proliferation due to decreased expression of *p21*. Nevertheless, *p21* expression was measured in all subsequent experiments as an established readout of p53’s transcriptional activity and because of its regulation by fasting (8). Interestingly, hepatic p53 protein was localized almost exclusively to the nuclear fraction, supporting the notion that hepatic p53 works primarily as transcriptional regulator and, as expected, was efficiently deleted by AAV-mediated Cre expression (Fig. 1*C*). We also found no indication that AAV’s *per se* affected hepatic p53 protein expression (Fig. S1, *A* and *B*), as shown previously for some cell types (15), underlining the suitability of our mouse model to dissect metabolic functions of p53 in the liver.

### Hepatic p53 is dispensable for metabolic homeostasis in the long term but required for the expression of p53 target genes in a diet-dependent manner

**Figure 2:**
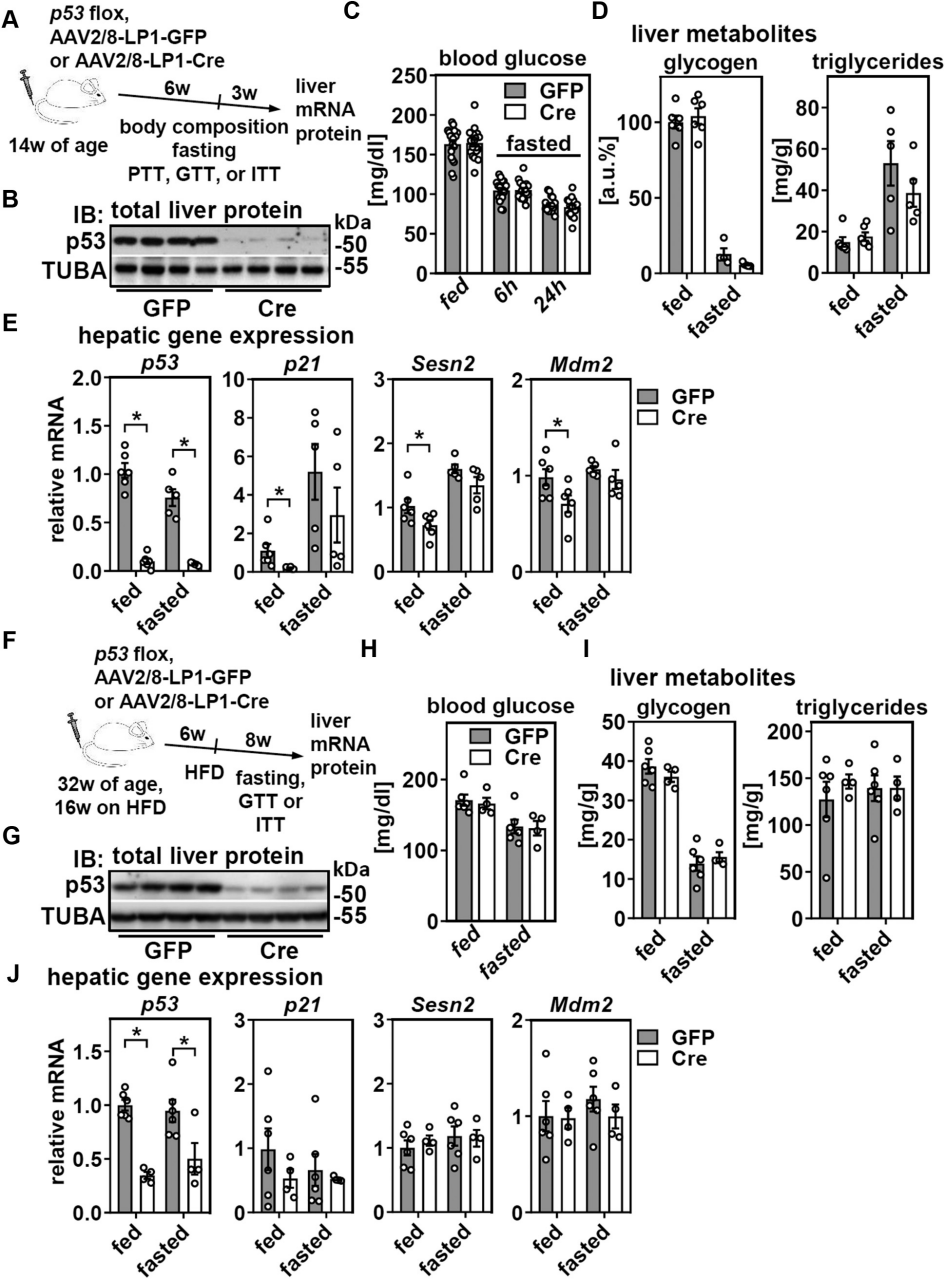
**Chronic deletion of hepatic p53 in adult mice does not affect metabolic homeostasis despite transcriptional responses in liver.** Male mice were treated as depicted in (*A*) and (*B*) hepatic p53 protein of *ad libitum*-fed mice analyzed by immunoblotting. TUBA served as loading control. *C*, blood glucose and (*D*) hepatic glycogen and triglyceride levels in *ad libitum*-fed or fasted mice were determined. *E*, expression of *p53* and its target genes were determined by qPCR. Male mice fed a HFD were treated as shown in (*F*) and (*G*) hepatic p53 protein of *ad libitum*-fed mice analyzed by immunoblotting. TUBA served as loading control. *H*, blood glucose and (*I*) hepatic glycogen and triglyceride levels in *ad libitum*-fed or 24 h fasted mice were determined. *J*, expression of *p53* and its target genes in liver were determined by qPCR. Data are represented as individual data points and mean ± SEM and ∗*p* < 0.05 *versus* mice with liver-specific GFP expression. HFD, high-fat diet.

Next, we induced deletion of *p53* in a bigger cohort of male mice, waited for 6 weeks, and metabolically characterized them within a period of 3 weeks (Fig. 2*A*). Despite an efficient deletion of p53 protein in liver until the end of the study (Fig. 2*B*), weight gain, body composition, and hepatic and circulating glucose levels did not differ between GFP and Cre-expressing mice (Figs. S2, *A*–*C* and 2*C*). Surprisingly, and in contrast to the consequences of short-term deletion of liver p53 (8), liver glycogen and triglycerides were also unchanged, irrespective of the feeding state (Fig. 2*D*). Moreover, pyruvate, glucose, and insulin tolerance were comparable between mice with and without hepatic p53 expression (Fig. S2, *D*–*F*). Finally, we analyzed hepatic gene expression and found that p53 deletion yielded the expected downregulation of its target genes *p21*, *sestrin 2* (*Sesn2*), and *mouse double minute 2 homolog* (*Mdm2*) without any alterations in *Ki67* expression (Figs. 2*E* and S2*G*). Notably, p53 deletion downregulated these genes in the *ad libitum*-fed state, whereas the fasting-induced upregulation of *p21* and *Sesn2* we reported previously (7, 8) was detectable even in the absence of hepatic p53 (Fig. 2*E*). Thus, although long-term deletion of p53 in liver induces alterations in hepatic gene expression, it does not affect metabolic homeostasis.

We then tested whether inducing p53 deletion in livers of HFD-fed, obese mice interferes with metabolic homeostasis (Fig. 2*F*). Knockout efficiency of hepatic p53 was slightly reduced (∼70% *versus* ∼90% in normal chow [NC]-fed mice, Fig. 2, *G* and *J*), potentially due to the longer experimental setup or because of an increased fraction of nonparenchymal cells, such as inflammatory cells, upon HFD feeding within the liver that also express p53. Notably, none of the mice with hepatocyte-specific deletion of p53, even after prolonged HFD feeding, developed macroscopically identifiable liver tumors (data not shown), suggesting that, at least in mice, additional oncogenic triggers are required for the formation of hepatocellular carcinomas (16). Body weight gain (Fig. S3*A*), blood glucose levels (*ad libitum*-fed and 24-h fasted), and hepatic glycogen and triglyceride concentrations did not differ between GFP and Cre-expressing mice (Fig. 2, *H* and *I*). Additionally, glucose and insulin tolerance were not affected by the deletion of p53 in liver (Fig. S3, *B* and *C*). Unexpectedly, loss of hepatic p53 failed to downregulate expression of its target genes, irrespective of the feeding state (Fig. 2*J*). Moreover, we observed a complete absence of the fasting-induced upregulation of *p21* and *Sesn2* in these mice (Fig. 2*J*), as observed in this (Fig. 2*E*) and all previous studies with NC-fed mice (7, 8). Hence, HFD feeding did not unravel metabolic aberrations upon p53 deletion in liver but, surprisingly, blunted the impact of both p53 deletion and fasting on the expression of p53 target genes.

### HFD abrogates the induction of hepatic p53 target genes by fasting without affecting p53 and disrupts FOXO1 signaling

**Figure 3:**
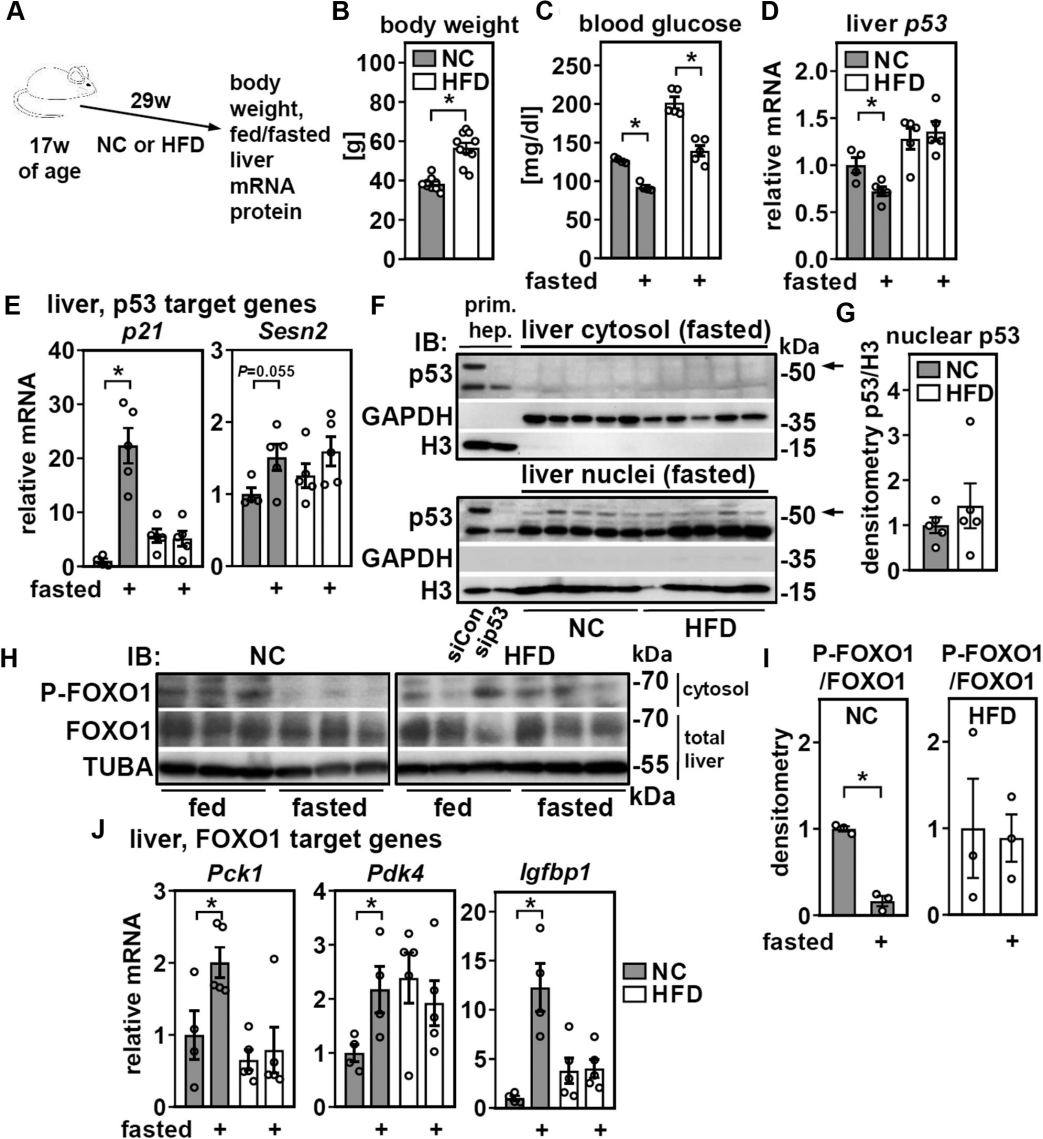
**High-fat diet (HFD) abrogates the induction of hepatic p53 target genes by fasting without altering protein expression or subcellular localization of p53 but associates with disrupted FOXO1 signaling.** Male mice were fed with normal chow (NC) or HFD as shown in (*A*) and (*B*), and their body weights determined. *C*, blood glucose was analyzed in *ad libitum*-fed or 24 h fasted mice. *D*, expression of *p53* and (*E*) its target genes in liver were determined by qPCR. *F*, liver tissue was processed into nuclear and cytosolic fractions and p53 protein analyzed by immunoblotting. Primary hepatocytes treated with siRNA targeting p53 served as band identity- and GAPDH and histone 3 (H3) as loading and fractionation controls. *G*, densitometric analysis of nuclear p53 protein. *H*, cytosolic fraction and total liver protein of NC and HFD-fed mice were analyzed by immunoblotting for the protein expression of phosphorylated and total FOXO1, respectively. *I*, densitometric analysis of phosphorylated FOXO1 protein. *J*, hepatic expression of known FOXO1 target genes in *ad libitum*-fed or 24 h fasted mice fed NC or HFD. Data are presented as individual data points and mean ± SEM and with ∗*p* < 0.05 *versus ad libitum*-fed mice. FOXO1, forkhead box O1 protein.

In order to dissect whether HFD feeding or aging as a consequence of prolonged HFD feeding is responsible for these effects, we subjected mice to the equivalent duration of either NC or HFD feeding (Fig. 3*A*). HFD feeding led to a ∼50% increase in body weight and, consistent with the induction of insulin resistance, higher blood glucose concentrations in both *ad libitum*-fed and fasted mice (Fig. 3, *B* and *C*). In liver, *p53* mRNA expression was downregulated by fasting in NC-fed mice, whereas HFD-fed mice tended to have higher p53 expression that was not affected by fasting (Fig. 3*D*). Strikingly, both *p21* and *Sesn2* lost their fasting-dependent induction in HFD-fed mice, with a much more pronounced effect on *p21* (Fig. 3*E*). We conclude that HFD-feeding, but not aging, is responsible for the lack of inducibility of *p21* and to a lesser extent *Sesn2*, by fasting. We therefore reasoned that HFD-feeding might interfere with p53 protein expression or localization because mRNA levels were not substantially changed (Fig. 3*D*). Contrary to this hypothesis, we found no evidence for HFD-induced alterations regarding the subcellular localization and/or abundance of hepatic p53 protein in fasted mice that could account for the differences in *p21* regulation (Fig. 3, *F* and *G*). These findings pointed to the involvement of transcriptional regulators other than p53. Indeed, an earlier study showed that p53 was not required for the upregulation of *p21* in livers of fasted mice and that a *p21* promoter-driven luciferase reporter in liver was less active in fasted mice that lacked FOXO1 or expressed a dominant-negative form of this transcription factor (17). This is also in accordance with the presence of FOXO binding sites in the p21 promoter (18). Hepatic FOXO1 activity is controlled by posttranslational modifications like phosphorylation that induce nuclear exclusion downstream of insulin signaling (19, 20, 21, 22, 23). Moreover, insulin resistance dampens the inhibitory effect of insulin on FOXO1 and leads to persistent nuclear FOXO1 activity (24, 25). We therefore analyzed expression and subcellular localization of FOXO1 in NC and HFD-fed mice. Hepatic *Foxo1* mRNA was not affected (Fig. S4), but we noticed a reduced feeding/fasting dynamic of phosphorylated FOXO1 in the cytosolic fraction of liver proteins in HFD-fed mice (Fig. 3, *H* and *I*), consistent with HFD-induced insulin resistance. Strikingly, known FOXO1 target genes (26) like *phosphoenolpyruvate carboxykinase 1* (*Pck1*), *pyruvate dehydrogenase kinase 4*, and in particular, *insulin-like growth factor-binding protein 1* (*Igfbp1*) mirrored this and showed a feeding/fasting dynamic in NC-fed mice, but not HFD-fed mice (Fig. 3*J*). In summary, HFD feeding disrupts the induction of p53 target genes by fasting without altering protein expression or subcellular localization of p53. Instead, *p21* mRNA regulation closely resembled the expression pattern of other FOXO1 target genes, suggesting that FOXO1 rather than p53 regulates hepatic *p21* expression in a context-dependent manner.

### Hepatic p53 expression is induced by refeeding and suppressed by FOXO1

**Figure 4:**
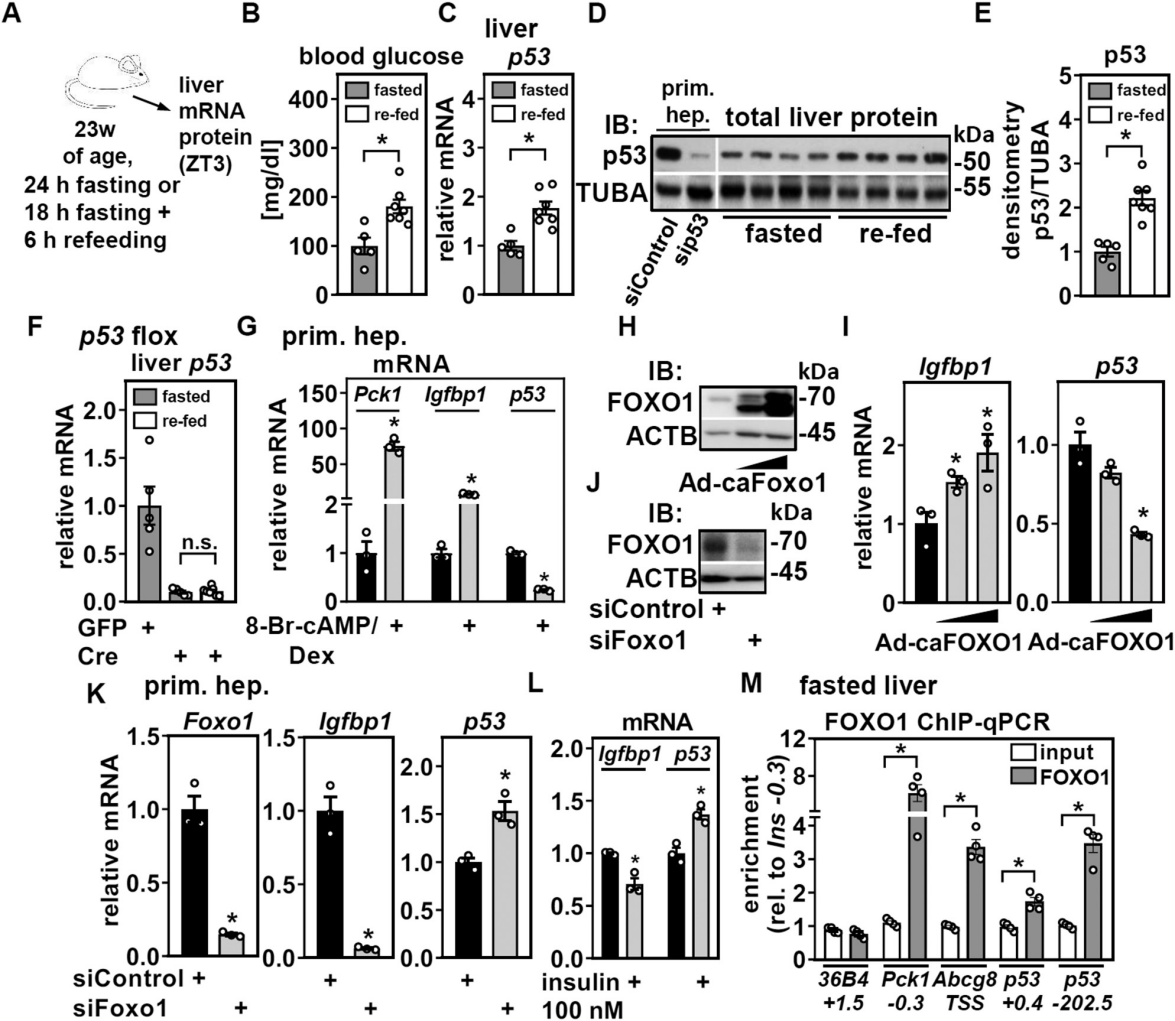
**Hepatic p53 mRNA and protein are induced by refeeding and regulated by FOXO1.** Male mice were treated as depicted in (*A*) and (*B*) blood glucose, (*C*) mRNA, and (*D*) protein expression of p53 determined. *D*, primary hepatocytes treated with siRNA targeting p53 served as band identity and TUBA as loading control (same membrane). *E*, densitometric analysis p53 protein in fasted and refed mice. *F*, mice were injected with AAV2/8 expressing GFP or Cre as indicated and 14 weeks later subjected to fasting or refeeding as shown in (*A*). Liver mRNA expression of *p53* was determined by qPCR. Primary mouse hepatocytes were (*G*) treated with 1 mM of a stable cAMP analog and 1 μM of dexamethasone (Dex) in serum-free DMEM lacking glucose and supplemented with 2 mM of pyruvate and 20 mM of lactate for 21 h and mRNA expression of *Pck1*, *Igfbp1*, and *p53* determined by qPCR. Hepatocytes were infected with increasing titers of an adenovirus expressing a constitutive-active (ca) FOXO1 and analyzed for (*H*) FOXO1 protein by immunoblotting and (*I*) mRNA expression of *Igfbp1* and *p53* by qPCR. Primary hepatocytes were treated with the indicated siRNA and 72 h later analyzed for (*J*) FOXO1 protein by immunoblotting. ACTB served as loading control. *K*, similarly treated hepatocytes were assessed for the mRNA expression of *Foxo1*, *Igfbp1*, and *p53* by qPCR. *L*, primary hepatocytes were incubated with 100 nM of insulin and mRNA expression of *Igfbp1* and *p53* determined by qPCR. *M*, FOXO1 binding at indicated sites was determined by chromatin immunoprecipitation (ChIP)-qPCR in fasted mouse liver. *36B4 +1.5* served as negative, whereas *Pck1 −0.3* and *Abcg8 TSS* served as positive controls. Data are presented as individual data points and mean ± SEM and with ∗*p* < 0.05 in (*B*, *C*, and *E*) *versus* fasted mice, in (*G*, *I*, *K*, and *L*) *versus* vehicle, siControl, or empty adenovirus treated hepatocytes, and in (*M*) *versus* input. DMEM, Dulbecco’s modified Eagle’s medium; FOXO1, forkhead box O1 protein; TSS, transcriptional start site.

We found that hepatic *p53* mRNA expression was reduced after 24 h of fasting (Figs. 2*E* and 3*D*), which is in accordance with previous findings (8). To substantiate this observation, we subjected mice to a fasting/refeeding cycle that usually maximizes food intake-dependent differences in gene expression. We compared mice after 18 h of fasting and a subsequent 6-h refeeding period to mice undergoing 24 h of fasting only, ending at the same zeitgeber time in order to exclude any circadian distraction (Fig. 4*A*). Refed mice showed the expected robust increase in blood glucose levels (Fig. 4*B*) and hepatic expression of feeding-associated genes such as *acetyl-CoA carboxylase 1* (*Acaca*) (Fig. S5). Moreover, p53 mRNA and protein expression in liver were induced, increasing ∼2-fold after 6 h of refeeding (Fig. 4, *C*–*E*). Primary hepatocytes depleted of p53 protein by siRNA served as a control to identify the specific p53 protein band, and an independent, similarly treated mouse cohort showed the same ∼2-fold induction upon refeeding (Fig. S6). The induction of p53 was blunted in mice with hepatocyte-specific deletion of p53 (Fig. 4*F*), showing that refeeding induces p53 expression in hepatocytes but not in other cell types that reside in liver.

We further analyzed p53 regulation in primary mouse hepatocytes. Depletion of carbohydrate response-element binding protein, a transcription factor activated by glucose metabolites and regulating hepatic gene expression upon refeeding (27), resulted in the expected loss of glucose responsiveness of its target gene, *Acaca*, whereas *p53* mRNA expression was not affected by either glucose or carbohydrate response-element binding protein knockdown (Fig. S7). On the other hand, a fasting-mimicking media containing a stable cAMP analog and dexamethasone decreased *p53* mRNA expression while inducing the positive control gene *Pck1* (Fig. 4*G*). Transcriptional regulation in liver upon fasting involves FOXO1 (28), and concordantly, we also found *Igfbp1* expression to be upregulated (Fig. 4*G*). We therefore tested whether ectopic FOXO1 expression would affect *p53* mRNA expression. Indeed, adenoviral expression of a constitutively active (ca) FOXO1 variant (Fig. 4*H*) that is insensitive to AKT-dependent phosphorylation and nuclear exclusion (29) showed the expected dose-dependent induction of *Igfbp1* and a clear downregulation of *p53* mRNA expression (Fig. 4*I*). FOXO1 depletion by siRNA (Fig. 4*J*) decreased *Igfbp1*, whereas *p53* was upregulated (Fig. 4*K*). Hence, FOXO1 represses *p53* transcription, which likely mediates the downregulation of *p53* in liver upon fasting and its induction upon refeeding, when FOXO1 is readily inactivated by rising insulin levels. In accordance, we found that insulin exposure increases *p53* mRNA expression in primary hepatocytes (Fig. 4*L*). In order to address FOXO1 binding near the *p53* gene locus, we performed chromatin immunoprecipitation (ChIP)-qPCR in fasted mouse liver. We found FOXO1 enriched at known binding sites near *Pck1* and *Abcg8* (30), validating the used antibodies for notoriously difficult FOXO1 ChIP in liver (Fig. 4*M*). Moreover, moderate FOXO1 binding could be detected downstream of exon 1 at *+0.4 kb* and more robust binding *−202.5 kb* upstream of the *p53* transcriptional start site (Fig. 4*M*) and in line with results from genome-wide analysis of FOXO1 binding in mouse liver (30). None of the genes located between *p53* and the FOXO1 binding at *−202.5 kb* were regulated by 24 h of fasting (7), suggesting that despite its distance also this site may be involved in the regulation of *p53* expression in liver.

### Acute loss of hepatic p53 alters glycogen and triglyceride accumulation and increases glycogen synthase phosphorylation

**Figure 5:**
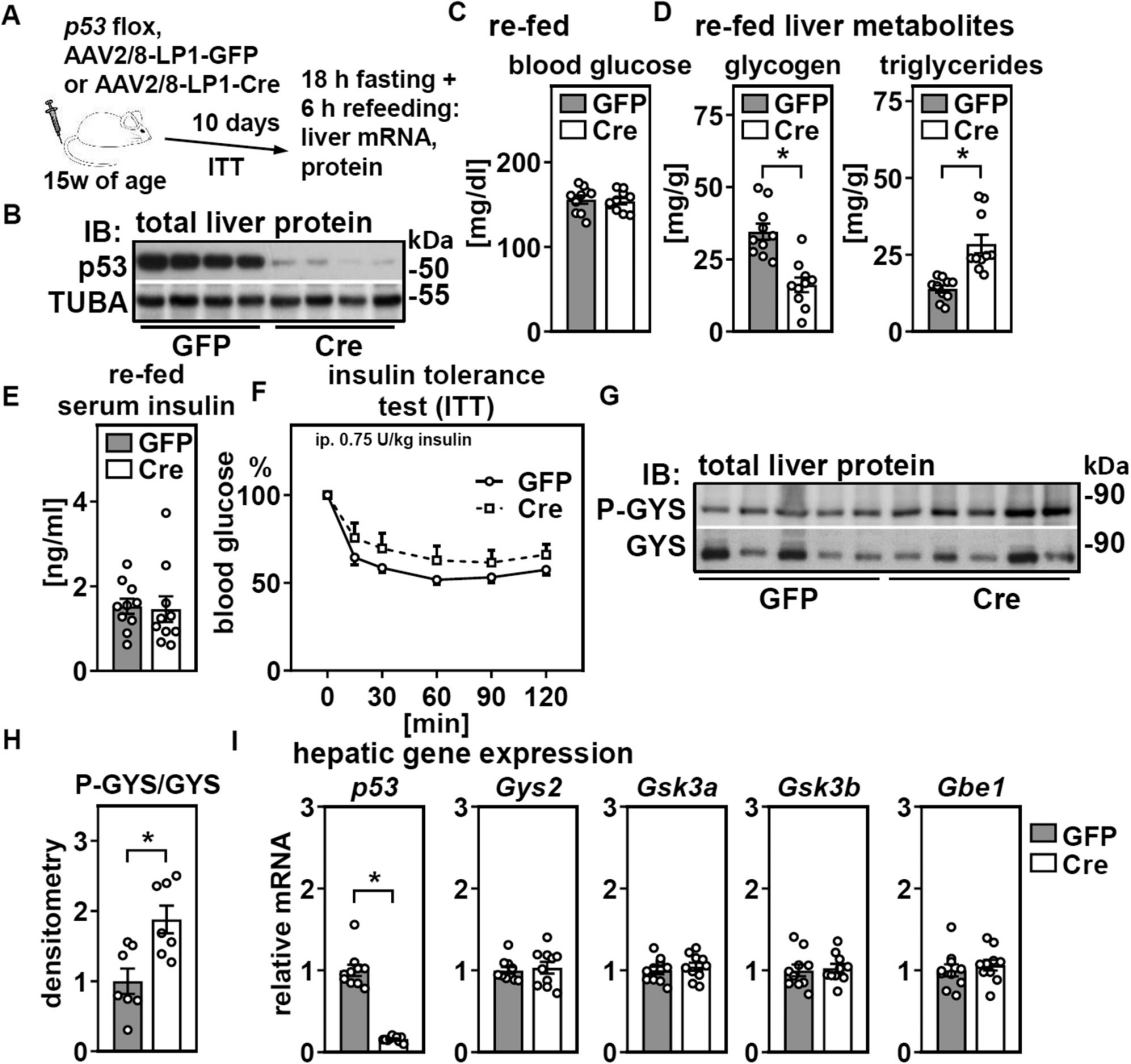
**Acute deletion of hepatic p53 in adult mice interferes with glycogen and triglyceride storage in liver upon refeeding.** Male mice were treated as depicted in (*A*) and (*B*) hepatic p53 protein of refed mice analyzed by immunoblotting. *C*, blood glucose and (*D*) hepatic glycogen and triglycerides in refed mice were determined. *E*, serum insulin levels of refed mice and (*F*) insulin tolerance were analyzed. *G*, phosphorylated glycogen synthase (GYS) in total liver protein of refed mice was determined by immunoblotting. *H*, densitometric analysis P-GYS protein in refed mice. *I*, hepatic mRNA expression of p53 and indicated genes related to glycogen synthesis in refed mice were analyzed by qPCR. Data are presented as individual data points and mean ± SEM or mean ± SEM only in (*F*) and with ∗*p* < 0.05 *versus* mice with liver-specific GFP expression.

Given the increase in hepatic p53 expression upon refeeding, we focused on the consequences of hepatic p53 deletion in refed mice, which is unexplored. We first determined the time-dependent deletion of hepatic p53 after injecting AAV, which gradually increased to more than 60% after 9 days (Fig. S8). Consistently, mice that were subjected to fasting/refeeding 10 days after the injection (Fig. 5*A*) showed efficient deletion of hepatic p53 protein (Fig. 5*B*). Refed blood glucose levels were unchanged in mice with liver-specific p53 deletion (Fig. 5*C*). However, hepatic glycogen was reduced, and triglycerides were increased (Fig. 5*D*), which is very similar to *ad libitum*-fed mice with acute p53 liver deletion (8). Serum insulin was unchanged and insulin responses comparable (Fig. 5, *E* and *F*), suggesting that insufficient insulin secretion or sensitivity are unlikely to be the underlying reasons for reduced glycogen accumulation. Because hepatic glycogen is nearly depleted in mice fasted for 16 h (31) and newly synthesized upon refeeding, we analyzed glycogen synthase (GYS) activity, which is regulated primarily by phosphorylation (32). Hepatic p53 deletion increased GYS phosphorylation in refed mice (Fig. 5, *G* and *H*), suggesting decreased synthase activity. No changes in the gene expression of *Gys2* or of known kinases that control GYS phosphorylation, such as *glycogen synthase kinase* (*Gsk*) 3 isoforms, or of *glycogen branching enzyme* (*Gbe1*) were observed (Fig. 5*I*).

### Acute loss of hepatic p53 impairs glycogen synthesis in a cell-autonomous manner

**Figure 6:**
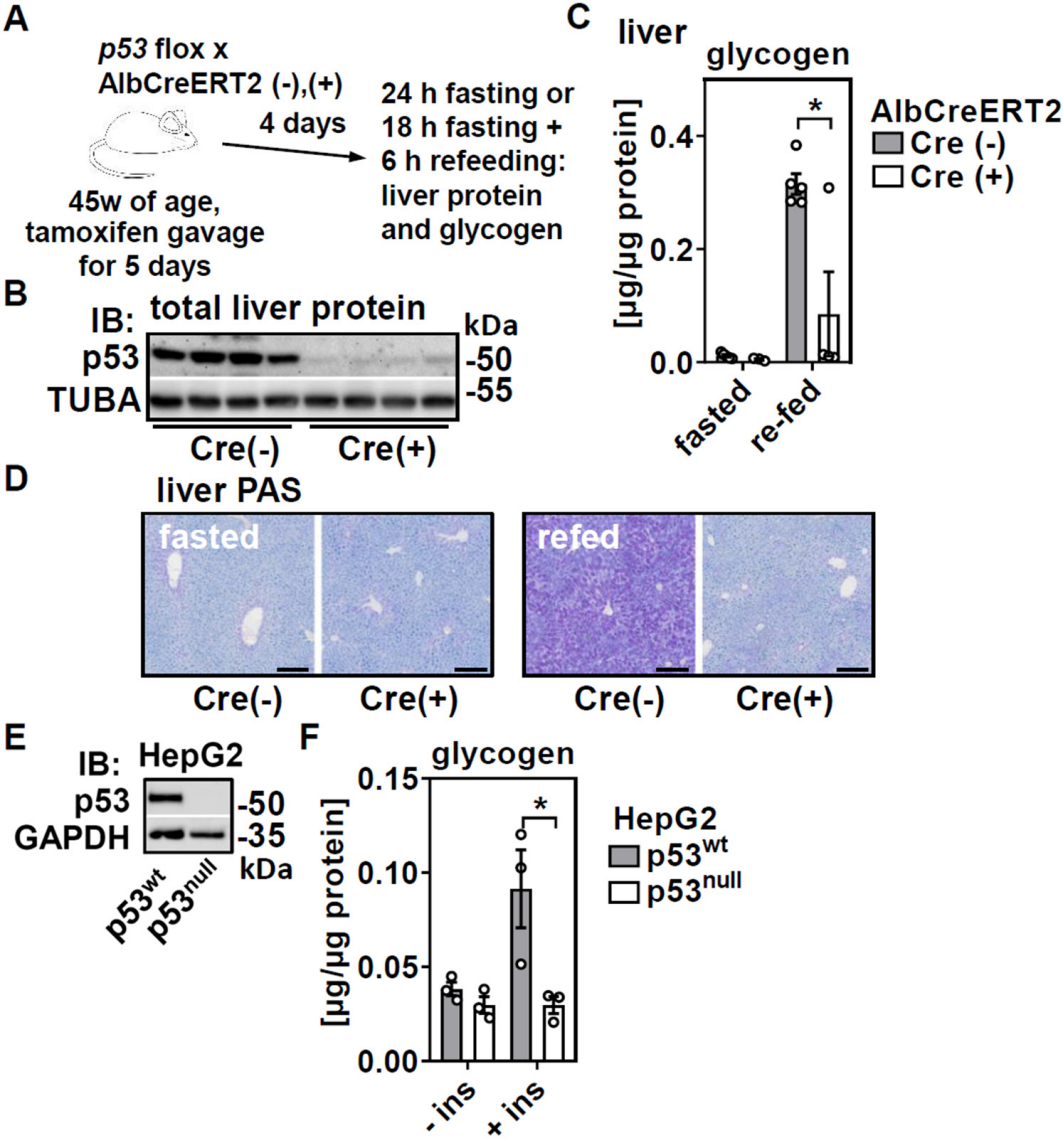
**Acute genetic deletion of hepatic p53 in adult mice blunts glycogen accumulation in liver upon refeeding in a cell-autonomous manner.** Male mice of the indicated genotypes were tamoxifen injected and treated as shown in (*A*) and (*B*) hepatic p53 protein of refed mice analyzed by immunoblotting. Hepatic glycogen levels in fasted and refed mice were determined (*C*) biochemically and (*D*) by Periodic acid-Schiff (PAS) staining in liver sections. *E*, human HepG2 cells with or without a CRISP/Cas9-mediated p53 deletion were analyzed by immunoblotting. GAPDH served as loading control. *F*, HepG2 were stimulated with 100 nM of insulin and glycogen content determined biochemically. Data are presented as individual data points and mean ± SEM and with ∗*p* < 0.05 *versus* (*C*) Cre (-) mice and (*F*) vehicle-treated cells. In (*D*), *black bars* equal 200 μm.

Another acute model of liver-specific p53 deletion (tamoxifen injection in *p53* floxed, AlbCreERT2 mice, Fig. 6*A*) with almost undetectable residual p53 protein (Fig. 6*B*) similarly showed reduced liver glycogen levels in refed mice (Fig. 6*C*). Periodic acid-Schiff staining of liver sections confirmed a uniform reduction of glycogen within periportal and pericentral hepatocytes in refed mice (Fig. 6*D*). We then treated human hepatoblastoma HepG2 cells with or without p53 expression (Fig. 6*E*) with insulin and found that the increase in cellular glycogen upon insulin stimulation was dependent on the presence of p53 (Fig. 6*F*), providing evidence for a hepatocyte-autonomous mechanism.

### Chronic loss of hepatic p53 induces a compensational activation of the glycogen synthesis pathway

**Figure 7:**
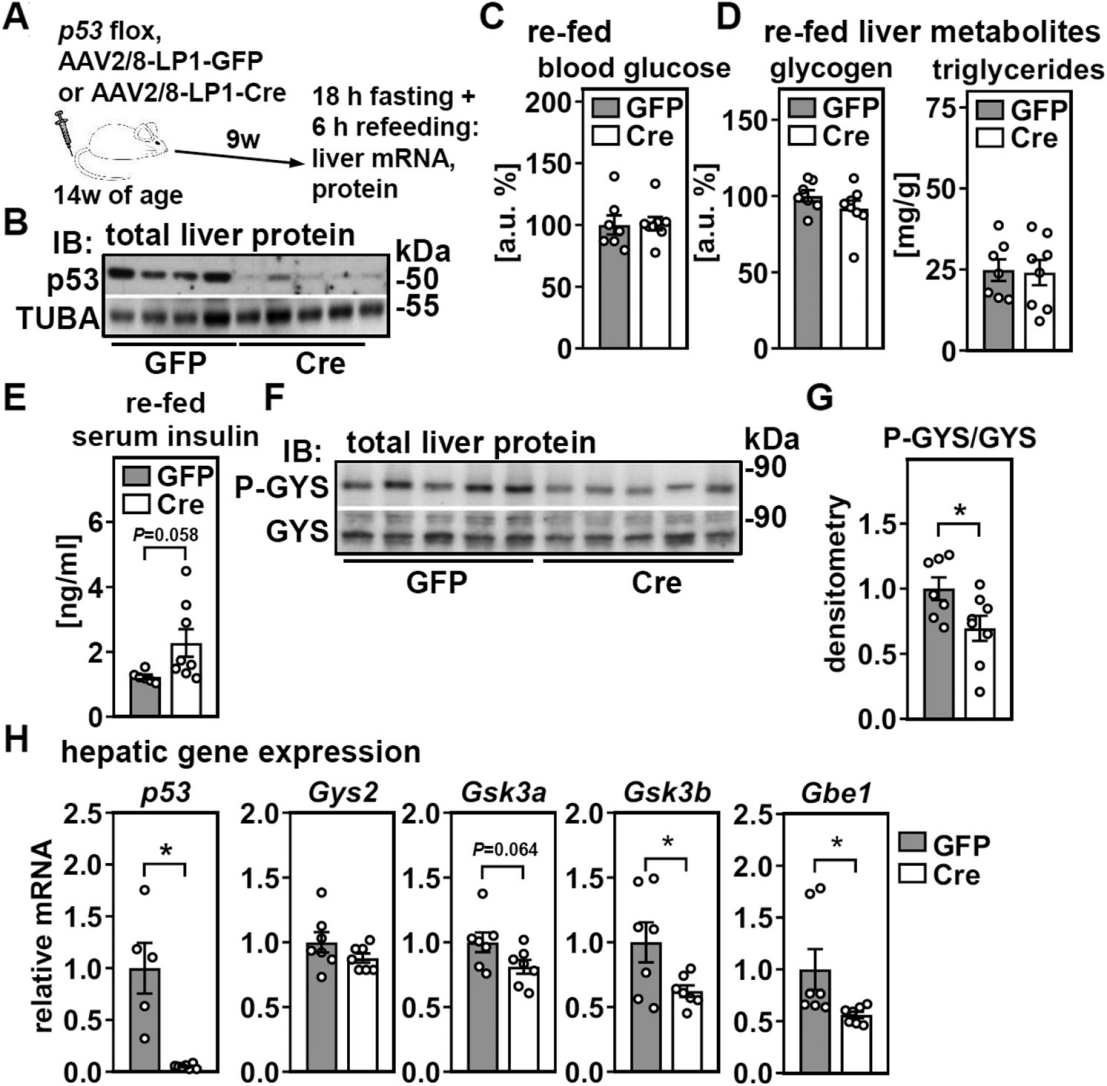
**Chronic deletion of hepatic p53 in adult mice induces a compensational activation of the glycogen synthesis pathway.** Male mice were treated as depicted in (*A*) and (*B*) hepatic p53 protein of re-fed mice analyzed by immunoblotting. *C*, blood glucose and (*D*) hepatic glycogen and triglycerides in re-fed mice were determined. *E*, serum insulin levels of re-fed mice were analyzed. *F*, phosphorylated glycogen synthase (GYS) in total liver protein of re-fed mice was determined by immunoblotting. *G*, densitometric analysis P-GYS protein in re-fed mice. *H*, hepatic mRNA expression of *p53* and indicated genes related to glycogen synthesis in re-fed mice were analyzed by qPCR. Data are presented as individual data points and mean ± SEM and with ∗*p* < 0.05 *versus* mice with liver-specific GFP expression.

Finally, we addressed whether long-term deletion of hepatic p53 would result in glycogen depletion and triglyceride accumulation, as observed in acute deletion models in refed mice. Therefore, instead of 10 days, mice were subjected to fasting/refeeding 9 weeks after AAV injection (Fig. 7*A*). Despite a comparable and efficient p53 deletion (Fig. 7*B*), there was no difference in any of the analyzed serum or liver metabolites (Fig. 7, *C* and *D*). However, refed serum insulin concentrations tended to be higher in mice that lacked p53 in liver (Fig. 7*E*), and GYS phosphorylation was decreased, implying increased glycogen synthesis (Fig. 7, *F* and *G*). Consistently, we found that mRNA expression of both *Gsk3* isoforms and that of *Gbe1* were decreased (Fig. 7*H*). We conclude that chronic loss of hepatic p53 does not affect glycogen or triglyceride levels in the liver but is associated with an increased activation of the glycogen synthesis pathway that may compensate for the reduced hepatic glycogen accumulation observed upon acute p53 deletion.

## Discussion

Whole-body p53 deficiency in mice is accompanied by metabolic abnormalities such as increased hepatic lipid accumulation (33, 34, 35, 36), which manifests before the mice die around 6 months of age due to the development of severe lymphomas or sarcomas (33, 37). Because p53 is expressed in many tissues that participate in the control of energy homeostasis, including relevant neurons in the brain (38), mouse models with cell-type–specific deletion of p53 are required to address the underlying mechanisms.

We therefore established a model of inducible p53 deletion in livers of adult mice *via* AAV2/8-mediated Cre expression. This model is, to our knowledge, the first AAV model to target liver p53 using a hepatocyte-specific promoter, providing efficient p53 deletion for months and lacking potential interference of an inflammatory response associated with the previously used adenoviral Cre expression (8).

Contrary to our expectations and to the effects of an acute deletion, we found that *ad libitum*-fed and fasted blood glucose, hepatic glycogen, and triglyceride levels were not affected by hepatic p53 knockout when analyzed 8 weeks after deletion induction. Moreover, pyruvate tolerance, as an indicator of gluconeogenic flux, as well as glucose and insulin tolerance were also unaltered, suggesting that, at least in the long term, hepatic p53 is dispensable for maintaining metabolic homeostasis in mice. This implies a metabolic flexibility to counterbalance the consequences of acute hepatic p53 deletion in the long term. The functional p53 homolog p63, shown to be induced by hepatic p53 deletion (33), was not responsible for this compensation since its mRNA levels were so low that we failed to reproducibly quantify its expression (C_t_ > 32, data not shown). p53 deletion in mice challenged with feeding a HFD yielded similar results, and no metabolic differences were observed between mice with and without p53 knockout in liver.

Unexpectedly, we found that HFD feeding abrogated the induction of p53 target genes upon fasting. This lack of induction was not due to altered expression or subcellular localization of p53 protein but was found to be associated with impaired FOXO1 regulation, a protein that is known to become persistently active upon HFD-induced insulin resistance (24, 25). Accordingly, there was a striking resemblance in expression pattern between FOXO1 and p53 target genes, particularly *p21*, which is in line with a previous report and the fact that p53 has a known genomic FOXO1 binding site in its promoter (17, 18). Thus, *p21* and possibly other p53 target genes may be under the control of both p53 and FOXO1 in a context-dependent manner. p53 appears to play a dominant role in livers of *ad libitum*-fed mice, whereas transcriptional control shifts to FOXO1 during fasting and HFD feeding, when FOXO1 is persistently active. This is supported by the observation that hepatic expression of p53 targets, such as *Mdm2* and *p21*, were downregulated by p53 deletion only in *ad libitum*-fed mice but not in fasted or HFD-fed mice.

Strikingly, in addition to the regulation of p53 target genes by FOXO1, we identify FOXO1 as a repressor of p53 expression, which likely accounts for the observed downregulation of hepatic *p53* mRNA upon fasting that, as one would expect, is blunted in insulin-resistant mice on an HFD. FOXO1 repressed *p53* mRNA expression in primary hepatocytes, providing evidence for a hepatocyte-autonomous regulation. In support of a direct regulation of *p53*, we show genomic FOXO1 binding at a proximal site and further upstream from its transcriptional start site and in accordance with previously published genome-wide FOXO1 binding in fasted mouse liver (30). Insulin upregulated *p53* mRNA expression in primary hepatocytes, presumably by FOXO1 inactivation and nuclear exclusion.

In further support of p53’s regulation by FOXO1, hepatic p53 mRNA and protein expression were induced by refeeding. Although refeeding only led to a modest 2-fold increase in the expression of p53 mRNA and protein, this induction was highly reproducible and likely to be physiologically relevant. Indeed, we found that mice lacking hepatic p53 acutely exhibited lower glycogen and higher triglyceride levels in liver upon refeeding. This was accompanied by reduced activation of GYS, suggesting that loss of p53 impairs glycogen synthesis. We also observed lower hepatic glycogen accumulation upon refeeding in another inducible and liver-specific p53-knockout mouse model, underlining the robustness of this effect. Moreover, human HepG2 cells accumulated less glycogen after insulin stimulation in the absence of p53, which suggests that this effect is not restricted to mouse cells. However, following refeeding, dysregulated glycogen and triglyceride levels in mice with p53 deletion were no longer detectable when analyzed after 2 months. This suggests that during refeeding, defects caused by p53 deletion are compensated for in the long term. Consistently, the glycogen synthesis pathway in livers of these mice was activated, which may account for this phenomenon.

Hepatic FOXO1 signaling has been previously linked to p53, by which p53-dependent activation of the NAD-dependent deacetylase, sirtuin 6, which deacetylates and inactivates FOXO1, resulted in the downregulation of FOXO1 targets like *Pck1* (39). In our model, and irrespective of feeding NC or HFD, *Pck1* expression was not affected by p53 deletion (Fig. S9, *A* and *B*), which is in contrast to other reports (40). Instead, we show that FOXO1 works as an upstream regulator of p53 expression in hepatocytes. Whether the downstream signaling of p53 and FOXO1 in liver is interdependent is currently unknown and will require further study. In cancer cells, p53 and FOXO share target genes (41); regarding FOXO3, direct interaction with p53 protein and transcriptional regulation of each other have been reported (42, 43, 44). Hence, the suggested regulatory network between p53 and FOXO regarding longevity and tumor suppression (41, 45) might very well extend to p53 and FOXO1 for controlling nutrient metabolism in liver. In this respect, it appears plausible that an activation of FOXO1 in liver, as observed during fasting, could negatively feed back on p53 expression to prevent exaggerated expression of targets, like *p21*, and its consequences for cell cycle progression.

Limitations of this study include the lack of *in vivo* evidence for the regulation of hepatic p53 by FOXO1 such as re-feeding mice that lack FOXO1 or multiple FOXOs that are expressed in liver (22). Moreover, we show that insulin treatment increases *p53* expression in primary hepatocytes, but whether this is dependent solely on FOXO1 has not been determined. Finally, although FOXO1 and not p53 was shown to confer the induction of p21 expression upon fasting (17), more experiments are needed to fully understand the relative contribution of p53 and FOXO1 to the context-specific regulation of p53 target genes in liver. These remaining aspects will be addressed by future studies.

**Figure 8:**
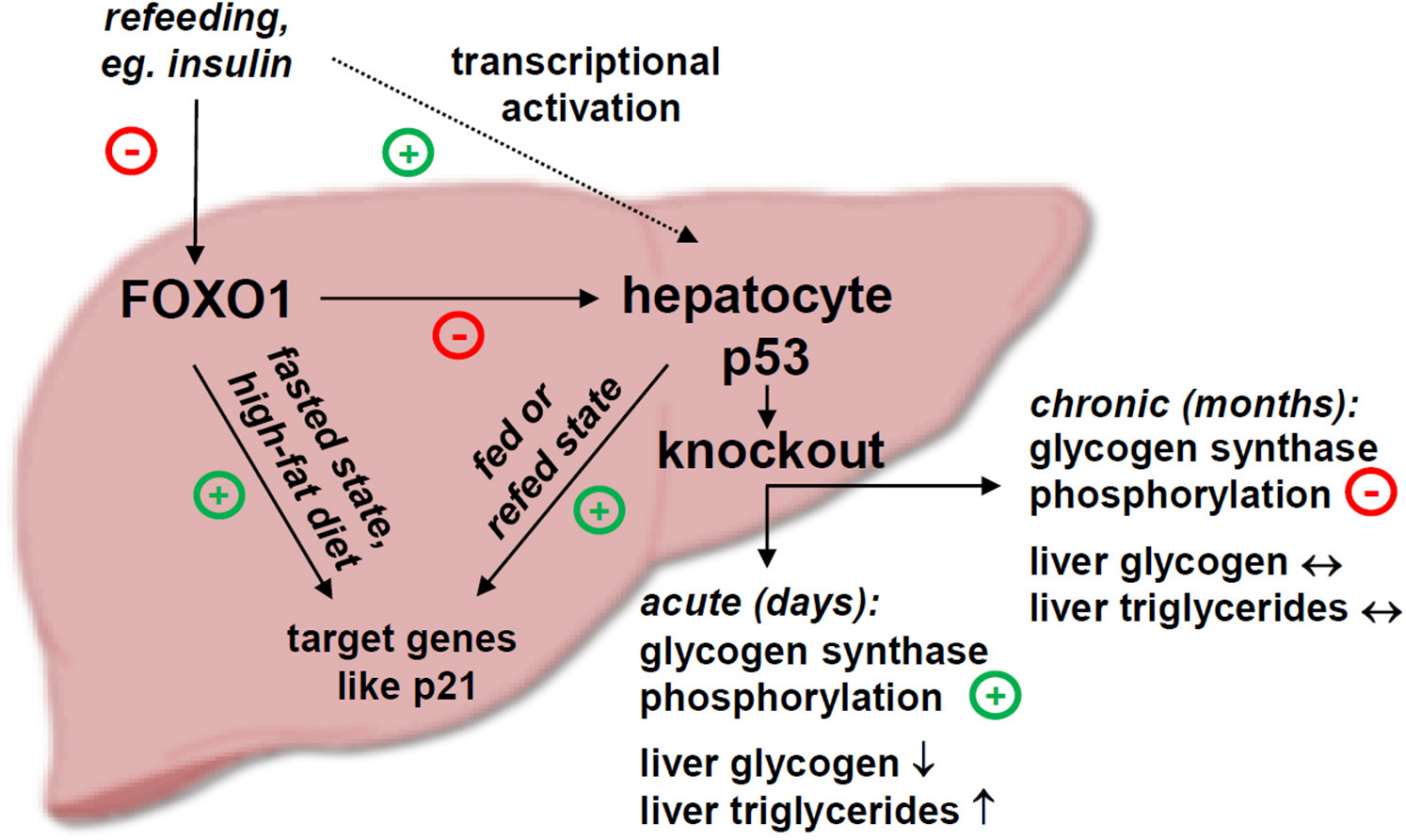
**Summary figure.** Liver p53 acutely regulates hepatic glycogen synthesis and triglyceride accumulation. Long-term deletion of p53 activates the glycogen synthesis pathway. FOXO1 represses hepatic p53 expression and, at least in part, mediates the upregulation of p53 upon refeeding. Both FOXO1 and p53 regulate target genes like *p21* in a context-dependent manner. FOXO1, forkhead box O1 protein.

Taken together, we show a role of p53 in the temporal control of triglyceride accumulation and glycogen synthesis in liver and identify hepatic FOXO1 as an upstream regulator of p53 target genes, as well as of p53 expression itself (Fig. 8). These findings have important implications for our understanding of the hepatic adaptation to nutrient availability.

## Experimental procedures

### Cloning, production, and purification of AAVs

Coral GFP or Cre cDNAs were amplified by PCR and cloned downstream of a Kozak sequence into the pds-AAV plasmids using *in-fusion* cloning (Clontech). Vectors were used to generate self-complementary AAV vectors, driven by the synthetic and highly hepatocyte-specific LP1 Promoter (11). AAV of the 2/8 serotype (AAV2rep, AAV8cap) were generated and titered as described previously (13). Purified AAV were dialyzed against 0.9% NaCl (ThermoFisher) and titers determined by qPCR, amplifying a LP1 promoter fragment (Table S1).

### Mouse studies and liver-specific p53 deletion

Animal procedures were approved by the corresponding authorities in Berlin/Germany and Graz/Austria. Sample size estimates were performed using G∗Power (46). Mice were on a C57BL/6J genetic background. Equal titers of AAV2/8 (1,25E+10 genomic copies) were injected *via* the tail vein into adult mice, fed either NC (ssniff R/M-H or 1320 fortified diet, Altromin) or HFD (60% kcal fat, D12492, ResearchDiets). p53 floxed mice were crossed with AlbCreERT2 mice (47) and received 100 mg/kg tamoxifen by oral gavage for 5 days.

### Metabolic characterization

Body composition was determined using NMR (Minispec LF50, Bruker). Blood glucose was measured using Contour strips (Bayer). Liver triglycerides were determined as previously described (48). To assess glucose tolerance, mice were intraperitoneally (ip.) injected with glucose (2 g/kg for lean, 0.5 g/kg for obese mice) after an overnight fast. Insulin tolerance was analyzed by ip. injection of insulin (0.5 U/kg for lean, 0.75 U/kg for obese mice) after a 4 h fast. Pyruvate tolerance was determined after an overnight fast and ip. injection of 2 g/kg pyruvate. For glycogen determination, ∼50 mg tissue or rabbit glycogen were hydrolyzed for 100 min at 100 °C in 1 M HCL and centrifuged at 12,000*g* for 30 min. The supernatant was neutralized using NaOH and glucose determined using G3293 reagent (Sigma-Aldrich).

### Gene expression

RNeasy columns (Qiagen) were used for RNA purification. cDNA synthesis and qPCR were performed as previously described (49). Gene expression levels were evaluated using standard curves, while *36b4* or *Hprt* served as housekeepers, and all primers are listed in Table S1.

### Immunoblotting

Immunoblotting was performed as before (50). Membranes were incubated with primary antibodies as listed in Table S2. A secondary antibody conjugated to horseradish peroxidase (Pierce) and a chemiluminescent kit was used for detection (ThermoFisher).

### Subcellular protein fractionation

Tissue was homogenized in hypotonic lysis buffer, incubated on ice for 45 min, and centrifuged at 12,000*g* for 15 min at 4 °C (=cytosol). The pellet was washed twice with PBS and resuspended in RIPA buffer (=nuclear fraction).

### Chromatin immunoprecipitation

ChIP was performed as described previously (51). In short, small pieces of frozen liver tissue from male mice fasted for 24 h were pulverized in liquid nitrogen and underwent cross-linking in 1% formaldehyde for 10 min, followed by quenching with 1/20 volume of 2.5 M glycine solution. Nuclear extracts were prepared by homogenizing in 20 mM Hepes, 0.25 M sucrose, 3 mM MgCl_2_, 0.2% NP-40, 3 mM β-mercaptoethanol, complete protease inhibitor tablet (Roche). Chromatin fragmentation was performed by sonication in 50 mM Hepes, 1% SDS, and 10 mM EDTA, using a Bioruptor (Diagenode) for 20 cycles of 30 s at the highest level. 75 μg of cross-linked and sonicated chromatin was immunoprecipitated overnight in 50 mM Hepes/NaOH at pH 7.5, 155 mM NaCl, 1.1% Triton X-100, 0.11% Na-deoxycholate, and complete protease inhibitor tablet, using 8 μg FOXO1 antibodies (ProteinTech 18592-1-AP and Abcam 39670, 4 μg each). Antibodies were precipitated with a 33% slurry of precleaned protein A Sepharose beads in 0.5% BSA/PBS for 2 h. Crosslinking was reversed overnight at 65 °C and DNA isolated using phenol/chloroform/isoamyl alcohol extraction. Enrichment of genomic sites in input and FOXO ChIP was determined by qPCR, normalized to a site near the *Ins* gene, and evaluated using standard curves for each primer pair (Table S1).

### NMR metabolomics

Quantification of circulating and hepatic glucose and liver glycogen in some experiments was performed by NMR metabolomics as previously described (8).

### Primary hepatocyte culture and treatment

Primary hepatocyte isolation and siRNA-mediated knockdown were performed as previously described (48). Hepatocytes were cultured in Dulbecco’s modified Eagle’s medium as indicated. siRNA oligonucleotides are listed in Table S1.

### HepG2 culture and treatment

HepG2 cells (ATCC) were cultivated in Dulbecco’s modified Eagle’s medium with 25 mM of glucose, supplemented with 4 mM glutamine, 10% FBS, and 500 U/ml penicillin-streptomycin (GIBCO) as previously (52). Glycogen was quantified using a kit (K646-100, BioVision). HepG2 p53 knockout was generated using CRISPR/Cas9 KO plasmids (sc-416469, SantaCruz) as described elsewhere (52).

### Liver histology

Liver histology and periodic acid-Schiff staining were performed as previously described (8).

### Statistics

Data are presented as individual data points and mean ± SEM. Representative results of at least three independent cell culture experiments are shown. Mouse cohorts were analyzed using two-tailed Student’s *t* tests or ANOVA as appropriate, and *p* < 0.05 was deemed significant.

## Data availability

All described data are contained within the manuscript.

## Supporting information

This article contains supporting information.

## Conflict of interest

The authors declare that they have no conflicts of interest with the contents of this article.
