# Supplementary material for: Hepatic p53 is regulated by transcription factor FOXO1 and acutely controls glycogen homeostasis
Source: J Biol Chem. 2022 Jul 20;298(9):102287. doi: 10.1016/j.jbc.2022.102287 (PMC9399478; doi:10.1016/j.jbc.2022.102287)
Supplement: Supplemental Figures S1–S9 and Tables S1, S2 [file mmc1.pdf]

Hepatic p53 is regulated by transcription factor FOXO1 and acutely controls glycogen homeostasis

Oster M *et al.*

ONLINE SUPPORTING INFORMATION

SUPPORTING INFORMATION FIGURE LEGENDS

Figure S1. AAV2/8 infection *per se* does not stabilize hepatic p53 protein. (A) Total liver protein of mice with or without AAV2/8 infection was analyzed for p53 protein by immunoblotting. Mice were AAV2/8 injected at an age of 10 weeks and analyzed 5 weeks later. Primary hepatocytes treated with siRNA targeting p53 served as band identity-, and RAN as loading control. (B) Densitometric analysis of p53 protein in livers of mice with or without AAV2/8 infection. In (B), data are presented as individual data points and mean ± sem.

Figure S2. Long-term deletion of hepatic p53 in adult mice does not affect metabolic homeostasis. Mice were treated as shown in Fig. 2A and (A) body weight gain of the final 7 weeks, (B) body composition, (C) liver glucose content, (D) pyruvate-, E) glucose-, and (F) insulin tolerance in GFP and Cre expressing mice determined. (G) Hepatic expression of *Ki67* was determined by qPCR. In (A-C,G), data are presented as individual data points and mean ± sem and in (D-F) as mean ± sem.

Figure S3. Long-term deletion of hepatic p53 in adult, high fat diet-fed mice does not affect body weight gain, glucose-, and insulin tolerance. Mice were treated as shown in Fig. 3A and (A) body weight gain within 8 weeks, (B) glucose-, and (C) insulin tolerance determined. In (A), data are presented as individual data points and mean ± sem, and in (B,C) as mean ± sem.

Figure S4. mRNA of *Foxo1* is not regulated by fasting in normal chow (NC) or high fat diet (HFD)-fed mice. Male mice were fed as shown in Fig. 4A and expression of *Foxo1* in liver determined by qPCR. Data are presented as individual data points and mean ± sem.

Figure S5. Refeeding induces anabolic gene expression in liver. Male mice were re-fed as depicted in Fig. 5A and mRNA expression of *Acaca* determined by qPCR. Data are presented as individual data points and mean ± sem and with *P<0.05 vs. fasted mice.

Figure S6. Hepatic p53 protein expression is induced by refeeding. Male mice were subjected to refeeding as depicted in Fig. 5A and (A) liver protein analyzed for p53 protrein expression by immunoblotting. Primary hepatocytes treated with siRNA targeting p53 served as band identity-, and TUBA as loading control. (B) Densitometric analysis of hepatic p53 protein in fasted and re-fed mice. In (B), data are presented as individual data points and mean ± sem and with *P<0.05 vs. fasted mice.

Figure S7. p53 expression in primary hepatocytes is not regulated by glucose and ChREBP. Primary murine hepatocytes were treated with control or ChREBP-targeting

siRNA overnight and 48 hours later exposed to 0.25 or 25 mM of glucose for 8 hours. Expression of indicated genes was analyzed by qPCR. Data are presented as individual data points and mean ± sem and with *P<0.05 vs. siControl treated cells.

Figure S8. AAV2/8-LP1 mediated Cre expression in liver deletes p53 expression within days. Male mice with floxed p53 alleles were tail vein-injected with AAV2/8 expressing GFP or Cre under the control of the liver-specific LP1 promoter. Hepatic p53 mRNA expression was analyzed at the indicated days after injection.

Figure S9. *Pck1* mRNA expression is not regulated by hepatic p53 knockout in livers of normal chow (NC) or high fat diet (HFD)-fed mice. (A) Hepatic *Pck1* expression in mice fed NC treated as shown in Fig. 2A was determined by qPCR. (B) Hepatic *Pck1* expression in mice fed HFD treated as shown in Fig. 3A was determined by qPCR. Data are presented as individual data points and mean ± sem.

SUPPORTING INFORMATION TABLES

Table S1. Oligonucleotide sequences (5'-3')

**qPCR**	m36B4 fw	TCATCCAGCAGGTGTTTGACA
(alphabetical)	m36B4 rv	GGCACCGAGGCAACAGTT
	mAcaca fw	GGTGAAGCTGGACCTAGAAGAGAA
	mAcaca rv	AAAGGCCAAACCATCCTGTAAGC
	mChrebpα fw	CGACACTCACCCACCTCTTC
	mChrebpα rv	TTGTTCAGCCGGATCTTGTC
	Cre	GCTTGCATGATCTCCGGTAT

	Cre	ATACCTGGCCTGGTCTGGA
	mFoxo1 fw	CTACGAGTGGATGGTGAAGAGC
	mFoxo1 rv	CCAGTTCCTTCATTCTGCACTCG
	mGbe1 fw	GGAGATTGACCCGTACCTGAA
	mGbe1 rv	ACATCTGTGGATGCCAAATGA
	cGFP	ATACCCTGGTGAATCGCATC
	cGFP	CATTGTGGGCGTTGTAGTTG
	mGsk3a fw	GGTGGCCATCAAGAAGGTT
	mGsk3a rv	CACAATATTGCAGTGGTCCAG
	mGsk3b fw	GCAGCCTTCAGCTTTTGGTA
	mGsk3b rv	ACTGACTTCCTGTGGCCTGT
	mGys2 fw	CCAACGATGCTGTCAGAAAA
	mGys2 rv	CCCTTCTATGAGCCATCTTCC
	mHprt	TGCTGACCTGCTGGATTACA
	mHprt	TATGTCCCCCGTTGACTGAT
	mIgfbp1 fw	ACGAGCACCTTGTTCAGCTC
	mIgfbp1 rv	GCAGCTGCTCCTCTGTCATC
	mKi67 fw	ATCATTGACCGCTCCTTTAGGT
	mKi67 rv	GCTCGCCTTGATGGTTCCT
	mMdm2 fw	AGCAGCGAGTCCACAGAGAC
	mMdm2 rv	ATCCTGATCCAGGCAATCAC
	mp21 fw	GACATTCAGAGCCACAGGCAC
	mp21 rv	GACCGAAGAGACAACGGCAC
	mp53 fw	GCTTCTCCGAAGACTGGATG
	mp53 rv	AATGTCTCCTGGCTCAGAGG
	mPck1 fw	ATCATCTTTGGTGGCCGTAG
	mPck1 fw	ATCATCTTTGGTGGCCGTAG
	mPdk4 fw	CTGCCTGACCGCTTAGTGA

	mPdk4 rv	CTTCTGGGCTCTTCTCATGG
	mSesn2 fw	CGCCACTCAGAGAAGGTTCA
	mSesn2 rv	ACGGGGTAGTCAGGTCATGT
**ChIP qPCR**	m36B4 +1.5 kb fw	CTGGGACGATGAATGAGGAT
	m36B4 +1.5 kb rv	AGCAGCTGGCACCTAAACAG
	mAbcg8 TSS fw	GCCAGAGTGTCCTTATCTCG
	mAbcg8 TSS rv	CTTTCTCCCAGCATTCCTC
	mIns -0.3 kb fw	CTTCAGCCCAGTTGACCAAT
	mIns -0.3 kb rv	AGGGAGGAGGAAAGCAGAAC
	mp53 +0.4 kb fw	TGATGTTAGGACCGACGAGC
	mp53 +0.4 kb rv	ACCCCTTAGGCCATCTCAGT
	mp53 -202.5 kb fw	GCTGGTCTTCACATCTGCCT
	mp53 -202.5 kb rv	GTTCACCTGCAGCACTTTGC
	mPck1 -0.3 kb fw	AGGTAACACACCCCAGCTAAC
	mPck1 -0.3 kb rv	GGCTCTTGCCTTAATTGTCAG
**AAV titering**	LP1 fw	GATCCCAGCCAGTGGACTTA
	LP1 rv	GTGCCTCACGACCAACTTCT
**siRNA**	siChrebp	UAU-UGA-ACC-GCC-UCU-UCU-G(UU)
	siControl	UUG-AUG-UGU-UUA-GUC-GCU-A(UU)
	siFoxo1	UUG-CUC-AUA-AAG-UCG-GUG-C(UU)
	sip53	GAA-UGA-GGC-CUU-AGA-GUU-A(UU)

Table S2. Antibodies

**antibody**	**product**	**application: immunoblotting**
p53	Cell Signaling #32532S (D2H90)	1:1000 in 5% BSA

FOXO1	Cell Signaling #2880S (C29H4)	1:500 in 5% BSA
p-FOXO1 (Thr24)	Cell Signaling #9464S	1:500 in 5% BSA
GYS	Cell Signaling #3886 (15B1)	1:500 in 5% BSA
p-GYS (Ser641)	Cell Signaling #3891S	1:500 in 5% BSA
TUBA	Cell Signaling #2144	1:2000 in 4% skim milk
GAPDH	Cell Signaling #2118 (14C10)	1:2000 in 4% skim milk
HISTONE H3	Sigma-Aldrich #06-755	1:2000 in 4% skim milk
ACTB	Santa Cruz #sc-47778	1:2000 in 4% skim milk
RAN	BD Transduction Laboratories # 610340	1:2000 in 4% skim milk
**antibody**	**product**	**application: ChIP**
FOXO1	Proteintech #18592-1-AP	4 µg / ChIP
FOXO1A	Abcam #ab39670	4 µg / ChIP

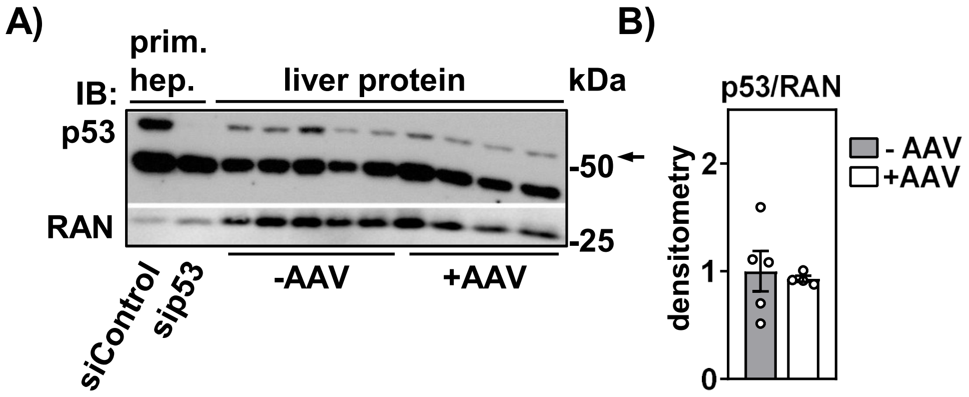

Figure S1. AAV2/8 infection *per se* does not stabilize hepatic p53 protein. (A) Total liver protein of mice with or without AAV2/8 infection was analyzed for p53 protein by immunoblotting. Mice were AAV2/8 injected at an age of 10 weeks and analyzed 5 weeks later. Primary hepatocytes treated with siRNA targeting p53 served as band identity-, and RAN as loading control. (B) Densitometric analysis of p53 protein in livers of mice with or without AAV2/8 infection. In (B), data are presented as individual data points and mean ± sem.

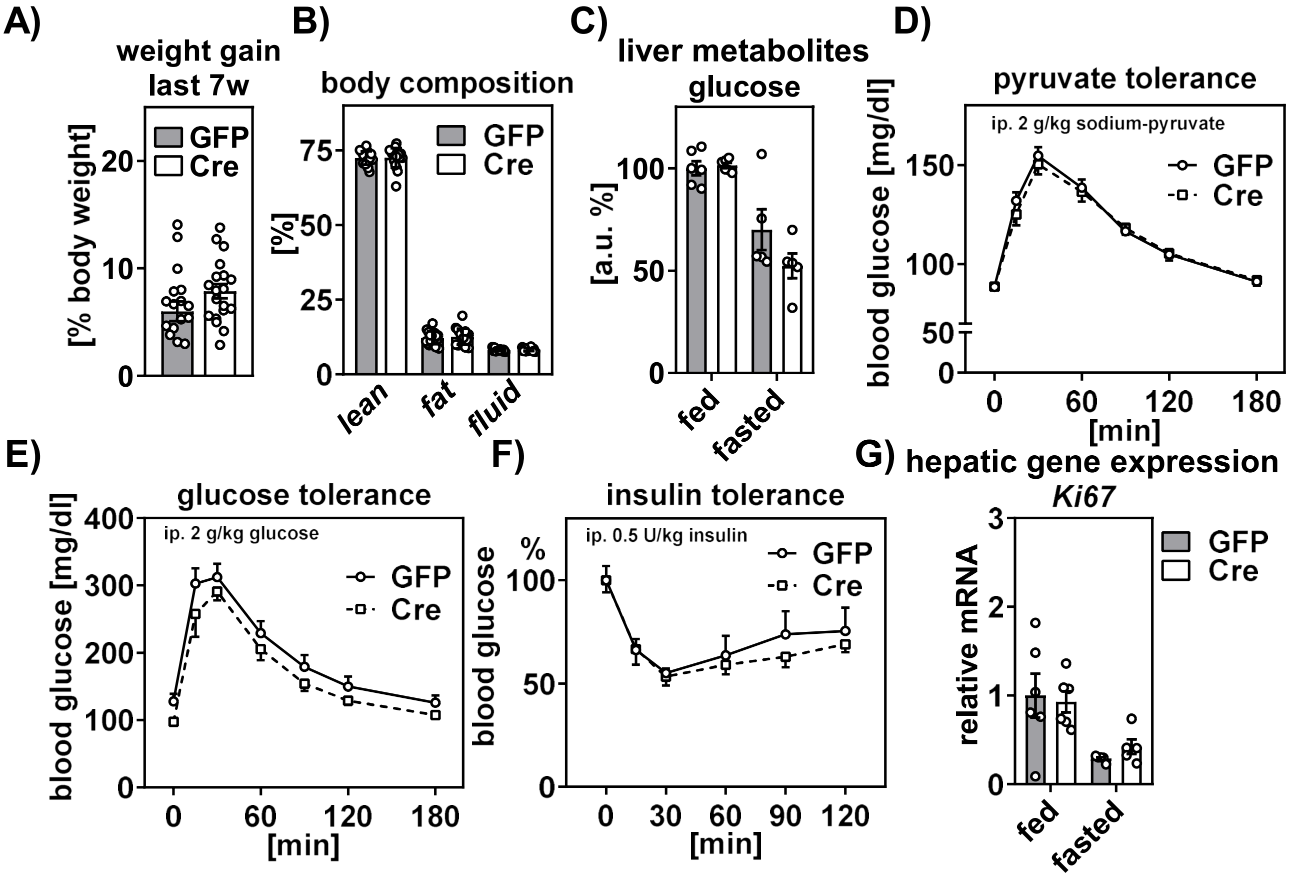

Figure S2. Long-term deletion of hepatic p53 in adult mice does not affect metabolic homeostasis. Mice were treated as shown in Fig. 2A and (A) body weight gain of the final 7 weeks, (B) body composition, (C) liver glucose content, (D) pyruvate-, E) glucose-, and (F) insulin tolerance in GFP and Cre expressing mice determined. (G) Hepatic expression of *Ki67* was determined by qPCR. In (A-C,G), data are presented as individual data points and mean ± sem and in (D-F) as mean ± sem.

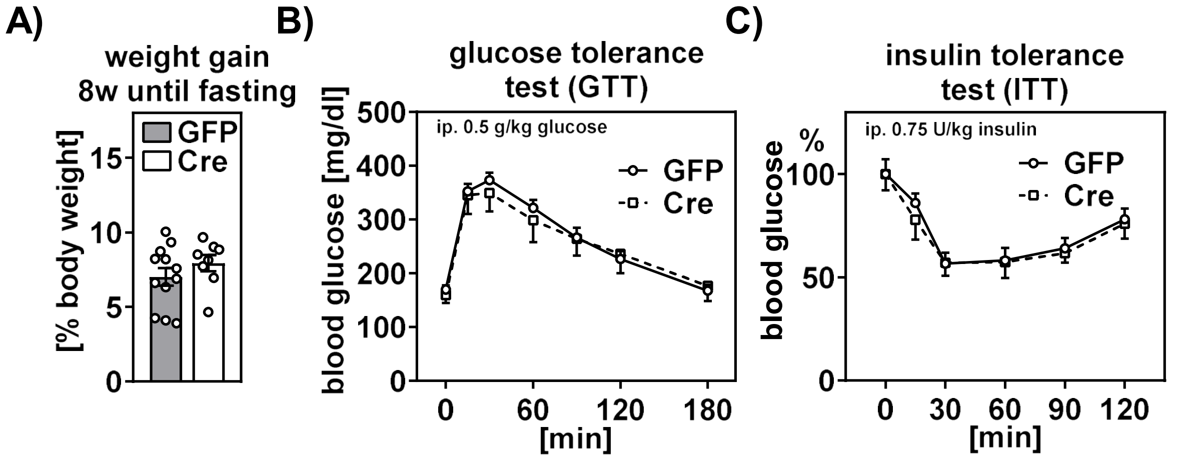

Figure S3. Long-term deletion of hepatic p53 in adult, high fat diet-fed mice does not affect body weight gain, glucose-, and insulin tolerance. Mice were treated as shown in Fig. 3A and (A) body weight gain within 8 weeks, (B) glucose-, and (C) insulin tolerance determined. In (A), data are presented as individual data points and mean ± sem, and in (B,C) as mean ± sem.

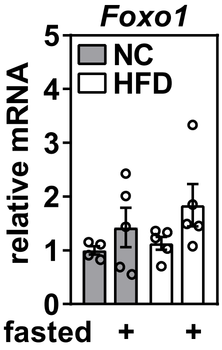

Figure S4. mRNA of *Foxo1* is not regulated by fasting in normal chow (NC) or high fat diet (HFD)-fed mice. Male mice were fed as shown in Fig. 4A and expression of *Foxo1* in liver determined by qPCR. Data are presented as individual data points and mean ± sem.

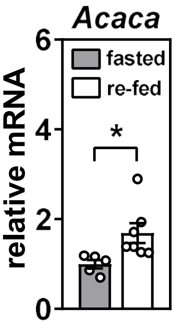

Figure S5. Refeeding induces anabolic gene expression in liver. Male mice were re-fed as depicted in Fig. 5A and mRNA expression of *Acaca* determined by qPCR. Data are presented as individual data points and mean ± sem and with **P*<0.05 vs. fasted mice.

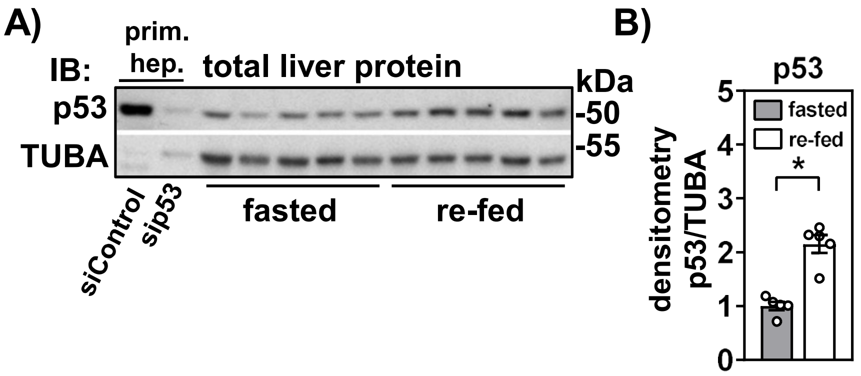

Figure S6. Hepatic p53 protein expression is induced by refeeding. Male mice were subjected to refeeding as depicted in Fig. 5A and (A) liver protein analyzed for p53 protrein expression by immunoblotting. Primary hepatocytes treated with siRNA targeting p53 served as band identity-, and TUBA as loading control. (B) Densitometric analysis of hepatic p53 protein in fasted and re-fed mice. In (B), data are presented as individual data points and mean ± sem and with *P<0.05 vs. fasted mice.

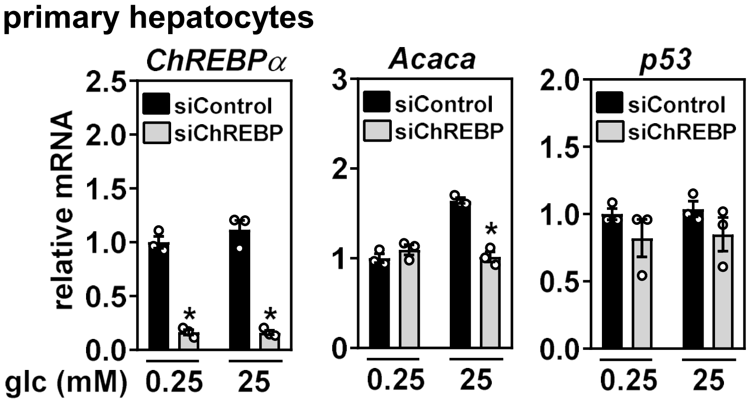

Figure S7. p53 expression in primary hepatocytes is not regulated by glucose and ChREBP. Primary murine hepatocytes were treated with control or ChREBP-targeting siRNA overnight and 48 hours later exposed to 0.25 or 25 mM of glucose for 8 hours. Expression of indicated genes was analyzed by qPCR. Data are presented as individual data points and mean ± sem and with *P<0.05 vs. siControl treated cells.

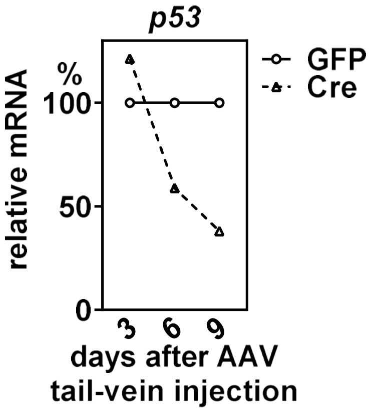

Figure S8. AAV2/8-LP1 mediated Cre expression in liver deletes p53 expression within days. Male mice with floxed p53 alleles were tail vein-injected with AAV2/8 expressing GFP or Cre under the control of the liver-specific LP1 promoter. Hepatic p53 mRNA expression was analyzed at the indicated days after injection.

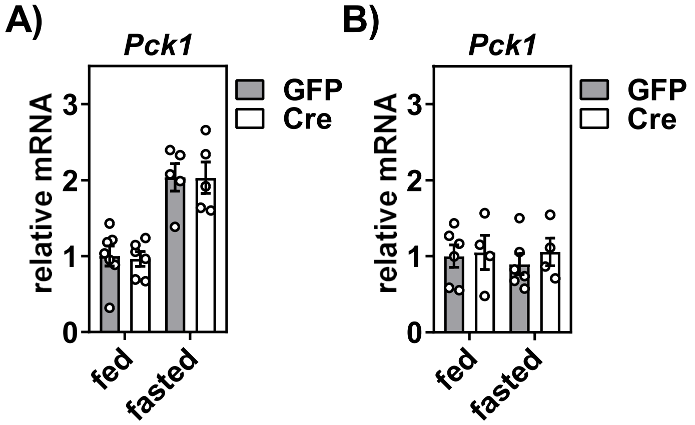

Figure S9. *Pck1* mRNA expression is not regulated by hepatic p53 knockout in livers of normal chow (NC) or high fat diet (HFD)-fed mice. (A) Hepatic *Pck1* expression in mice fed NC treated as shown in Fig. 2A was determined by qPCR. (B) Hepatic *Pck1* expression in mice fed HFD treated as shown in Fig. 3A was determined by qPCR. Data are presented as individual data points and mean ± sem.
